# Assessment of Electronic Sensing Techniques for the Rapid Identification of Alveolar Echinococcosis through Exhaled Breath Analysis

**DOI:** 10.3390/s20092666

**Published:** 2020-05-07

**Authors:** Andrzej Kwiatkowski, Tomasz Chludziński, Tarik Saidi, Tesfalem Geremariam Welearegay, Aylen Lisset Jaimes-Mogollón, Nezha El Bari, Sebastian Borys, Benachir Bouchikhi, Janusz Smulko, Radu Ionescu

**Affiliations:** 1 Department of Metrology and Optoelectronics, Faculty of Electronics, Telecommunications and Informatics, Gdańsk University of Technology, 80233 Gdańsk, Poland; Andrzej.Kwiatkowski@pg.gda.pl (A.K.); tomekchlud@gmail.com (T.C.); janusz.smulko@pg.edu.pl (J.S.); 2 Sensor Electronic & Instrumentation Group, Faculty of Sciences, Department of Physics, Moulay Ismaïl University of Meknes, B.P. 11201, Zitoune, Meknes 50050, Morocco; saidi.tareq@gmail.com (T.S.); benachir.bouchikhi@gmail.com (B.B.); 3 Biotechnology Agroalimentary and Biomedical Analysis Group, Faculty of Sciences, Department of Biology, Moulay Ismaïl University of Meknes, B.P. 11201, Zitoune, Meknes 50050, Morocco; n.elbari@umi.ac.ma; 4 Department of Electronics, Electrical and Automatic Engineering, Rovira i Virgili University, 43007 Tarragona, Spain; gem.tesfa@gmail.com; 5 The Ångström Laboratory, Division of Solid State Physics, Department of Materials Science and Engineering, Uppsala University, 75121 Uppsala, Sweden; 6 GISM Group, Faculty of Engineering and Architecture, University of Pamplona, Pamplona 543050, Colombia; lissetjaimes@gmail.com; 7 Department of Chemical Engineering, Complutense University of Madrid, 28040 Madrid, Spain; 8 University Centre of Maritime and Tropical Medicine, 81519 Gdynia-Redlowo, Poland; sebek07@op.pl

**Keywords:** echinococcosis, diagnosis, breath analysis, chemical gas sensors, DC resistance measurements, AC fluctuation measurements

## Abstract

Here we present a proof-of-concept study showing the potential of a chemical gas sensors system to identify the patients with alveolar echinococcosis disease through exhaled breath analysis. The sensors system employed comprised an array of three commercial gas sensors and a custom gas sensor based on WO_3_ nanowires doped with gold nanoparticles, optimized for the measurement of common breath volatile organic compounds. The measurement setup was designed for the concomitant measurement of both sensors DC resistance and AC fluctuations during breath samples exposure. Discriminant Function Analysis classification models were built with features extracted from sensors responses, and the discrimination of alveolar echinococcosis was estimated through bootstrap validation. The commercial sensor that detects gases such as alkane derivatives and ethanol, associated with lipid peroxidation and intestinal gut flora, provided the best classification (63.4% success rate, 66.3% sensitivity and 54.6% specificity) when sensors’ responses were individually analyzed, while the model built with the AC features extracted from the responses of the cross-reactive sensors array yielded 90.2% classification success rate, 93.6% sensitivity and 79.4% specificity. This result paves the way for the development of a noninvasive, easy to use, fast and inexpensive diagnostic test for alveolar echinococcosis diagnosis at an early stage, when curative treatment can be applied to the patients.

## 1. Introduction

Human Alveolar Echinococcosis (AE) is a pathogenic zoonotic disease produced by the infection with the larval form of the fox tapeworm *Echinococcus multiloculari*. AE is confined to the northern hemisphere, in particular to regions of China, Russia, Europe and North America [1,2,3]. Although AE is considered a rare disease, with average incidences between 0.03 and 0.30 cases per 100,000 inhabitants per year, currently there is an increased trend in its incidence [3]. In Europe for instance, the disease expanded in the last two decades from Central Europe to regions from Northern, Eastern and Western Europe [3], while in China the prevalence is currently higher than 5% [4]. 

AE manifests itself as a silently-progressing hepatic disorder in humans, and is characterized by an asymptomatic incubation period of 5–15 years, with slow development of a primary tumor-like lesion that is usually located in the liver [1]. If left untreated, the disease is progressive and fatal, but an early diagnosis promoting radical surgery of the lesion followed by suitable drug treatment can be curative [1]. 

Considering that the early stages of infection are usually asymptomatic, the diagnosis of AE may often be incidental, associated with imaging investigations performed for other clinical causes. The disease is detected by radiography, ultrasound or other imaging techniques such as computed tomography scans (CT) or magnetic resonance imaging, combined with case history. Depending on the symptoms and clinical signs, other complementary investigations can be realized for disease confirmation, such as serology tests, enzyme-linked immunosorbent assay (ELISA), immuno-blotting or biopsy [5]. All these methods are laboratory techniques that require qualified operators and are time consuming (several hours up to a few days to obtain the result).

Unfortunately, in many patients the disease is diagnosed at an advanced stage [1], therefore an alternative diagnostic method that could allow for an early diagnosis is highly desirable. Exhaled breath analysis holds great potential in this regard, because it reflects changes in the metabolism produced by the disease at an early stage [6,7]. These changes can be conveniently detected with electronic nose systems comprising chemical gas sensors, which are trained to recognize a pattern of volatile organic compounds (VOCs) that corresponds to the disease [8,9,10]. This method holds important advantages over the standard diagnosis methods, such as noninvasiveness (in contrast with a blood test or liver biopsy), easiness of use (does not require difficult training), portability (onsite method), fast results (several minutes) and low cost (estimated price of a test is around 10 euros).

Both commercial Taguchi type gas sensors developed for VOCs detection as well as many types of nanomaterial-based chemical gas sensors (e.g., nanoparticles and nanowires of different materials, carbon nanotubes, carbon black filaments, conducting polymers, etc.) were successfully employed in electronic nose systems developed for breath analysis [11,12,13]. DC sensing measurements, where the change in sensors resistance at constant voltage operation is analyzed, are commonly employed. Although less common, AC fluctuation of sensors resistance, which utilizes low-frequency noise (flicker noise) as a source of information, can provide a higher level of information [14].

In a very recent study, it was shown that a specifically-designed electronic nose system based on organically-functionalized metal nanoparticles trained for the detection of AE pattern in exhaled breath can discriminate with high accuracy between AE patients and healthy controls [15]. In that study, the sensors were operated in the DC mode. In the present study, we assess the performance of both DC and AC sensing methods for the diagnosis of AE from exhaled breath analysis, on a patient cohort completely independent of the one reported in [15]. For performing this study, we selected an array of Taguchi type chemical gas sensors that present a priori important features associated with AE metabolism compounds, and a novel custom gas sensor based on WO_3_ nanowires doped with gold nanoparticles that was recently reported for breath sensing and can further favor breath VOCs detection [16]. 

## 2. Materials and Methods

### 2.1. Patients and Samples Collection

The study protocol and procedures were approved by the Independent Bioethical Commission for Science Research at the Medical University of Gdańsk, Poland (ethical approval code: NKBBN/471/2014). All volunteers signed the informed consent before being included in the study. Only adult volunteers participated in the study. The personal data collected for the purpose of this study were anonymized by applying the aggregation and K-anonymity anonymization techniques.

In the study there were included 11 adult patients diagnosed with AE at the Clinic of Tropical and Parasitic Diseases from Gdynia, Poland, and a control group of 6 volunteers selected among the medical staff of the clinic. For AE confirmation, several tests were performed: serological tests (ELISA, Western blot), analysis of DNA samples by Polymerase Chain Reaction (PCR), and microscopic detection of parasites. Information about all volunteers is provided in Table 1.

The patients were admitted to the hospital at least one day before their breath was sampled. The patients did not do any physical activity during the hospitalization, and all volunteers did not eat or smoke during the day and the night prior to breath sampling. All samples were collected in the same room of the Clinic for avoiding differences in the matrix of exogenous compounds inhaled by the volunteers, during the interval from 8 am to 10 am for avoiding artefacts due to intra-day variations of breath composition [18].

For each volunteer two breath samples were collected employing the BioVOC^TM^ sampler (Markes International, UK). Briefly, the volunteer inhaled air from the environment and exhaled it normally through a disposable mouthpiece into the BioVOC until emptying the lungs, during approximately 10 s. The first part of the breath exited through the open end without return of the 129 mL volume BioVOC sampler, which retained inside only the last portion of the breath. This corresponds to the end-tidal breath that contains the metabolites exchanged by the blood with the lungs, where disease-specific VOCs can be found [6]. By means of a plunger, the breath retained inside the BioVOC was slowly and steadily pushed during 10 s into a storage glass tube containing a hydrophobic material suitable for breath VOCs preconcentration that prevents the retention of breath moisture (ORBO 420 Tenax^®^ TA sorption tube, Sigma-Aldrich, Spain), which was connected to the open end of the BioVOC. For increasing the concentration of the breath VOCs preconcentrated by the sorbent material, in each sorbent tube two breath samples provided by the same volunteer were acquired. The sorbent tubes were stored at 4 ºC in a refrigerator for biomedical samples before analysis, which was performed within four months of when the samples were collected. 

Following manufacturer indications, for cleaning purposes and for avoiding cross-contamination between the breath of different volunteers, before every use the BioVOC was disassembled into its constituent parts, which were introduced for 15 min in a solution of 20 mL of disinfectant (Milton, France) dissolved in 1 L of distilled water, and left to naturally dry without wiping.

### 2.2. Gas Sensors System

The breath samples were analyzed with a sensing system comprising four resistive gas sensors:

- **S1:** TGS8100 sensor (commercial sensor acquired from Figaro USA, Inc), optimized for target gases such as ethanol and hydrogen.
- **S2** and **S3**: Two MiCS-6814 sensors (commercial sensors acquired from SGX Sensortech Limited, UK), containing different gas-sensitive layers optimized for NH_3_ and CO detection, respectively. S2 also detects other gases such as ethanol, hydrogen, propane and iso-butane, and S3 gases such as H_2_S, ethanol, hydrogen, ammonia, methane, propane and iso-butane.
- **S4**: Custom sensor based on a ~159 nm thick WO_3_ nanowires layer doped with gold nanoparticles, fabricated by catalyst free, relatively low temperature single-step Aerosol Assisted Chemical Vapor Deposition (AACVD). Detailed information on the fabrication and characterization of this sensor can be found elsewhere [16].

### 2.3. Sensing Measurements

**Figure 1:**
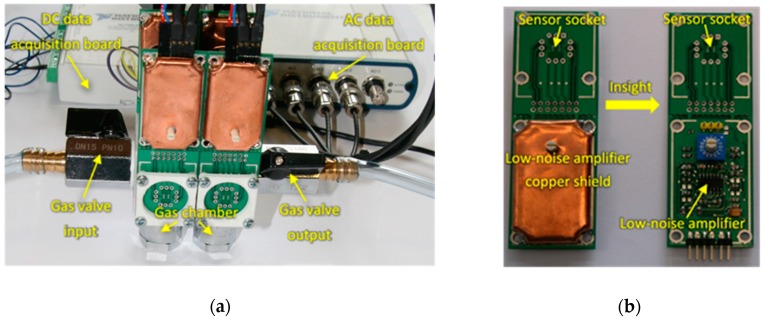
(**a**) Measurement setup; (**b**) Insight of the AC fluctuations measurement board.

For performing the sensing measurements, an experimental setup was designed for samples measurement employing two different sensing techniques that allowed for the concomitant acquisition of both sensors DC resistance changes upon exposure to the breath samples, as well as the AC fluctuations through the sensors during breath samples exposure (Figure 1).

**Figure 2:**
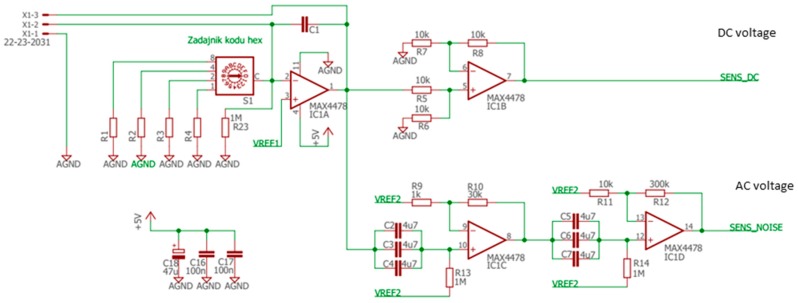
Electronic circuit.

The sensors were placed inside a tiny gas-sensing test chamber (20 mL volume), to which the electronic circuit developed for DC resistance and AC fluctuations measurement shown in Figure 2 was attached. This electronic circuit was designed to record the voltage across the gas sensors placed in the feedback loop of the low-noise operational amplifier MAX4478 (Maxim Integrated, San Jose, CA, USA). This setup filters out the DC and AC voltage components into two separate channels, which allows for the concomitant measurement of the DC resistance and AC fluctuation across the sensors.

Samples measurement followed the sequence indicated in Table 1, measuring first one sample provided by each volunteer and then the second sample of each volunteer. During the measurements, the commercial sensors were operated at the working temperatures specified by the producer, and the WO_3_ nanowires sensor was operated at the optimized temperature of 160 °C [16]. Samples measurement procedure consisted of the following steps:

1. Opening the test chamber and cleaning it with synthetic air flow (100 mL/min flow-rate) for at least 25 min.
2. Closing the test chamber and waiting approximately 30 min for the stabilization of sensors baseline.
3. Measuring DC and AC components by recording the voltage in independent channels for each sensor.
4. Producing vacuum, up to 420 mmHg, for approximately 20 sec in the test chamber by means of an external pump (model SAILFO 1A50361), just before injecting the breath VOCs sample.
5. Injecting 20 mL of breath VOCs sample in the test chamber by means of a commonly used disposable medical gas syringe (Becton-Dickinson Discordit II model). To this end, the sorbent Tenax TA material storing the breath sample was previously introduced in a sealed glass vial (20 mL volume) provided with a septum, which was heated at 200 ºC for 15 min in an oven. This produced the thermal desorption of the absorbed breath VOCs, which were captured by the gas syringe by introducing its needle through the septum, and then injected into the sensors test chamber.
6. Waiting ~30 min for the stabilization of sensors responses.
7. Measuring the DC and AC components by separately recording the voltages for the different sensors.

**Figure 3:**
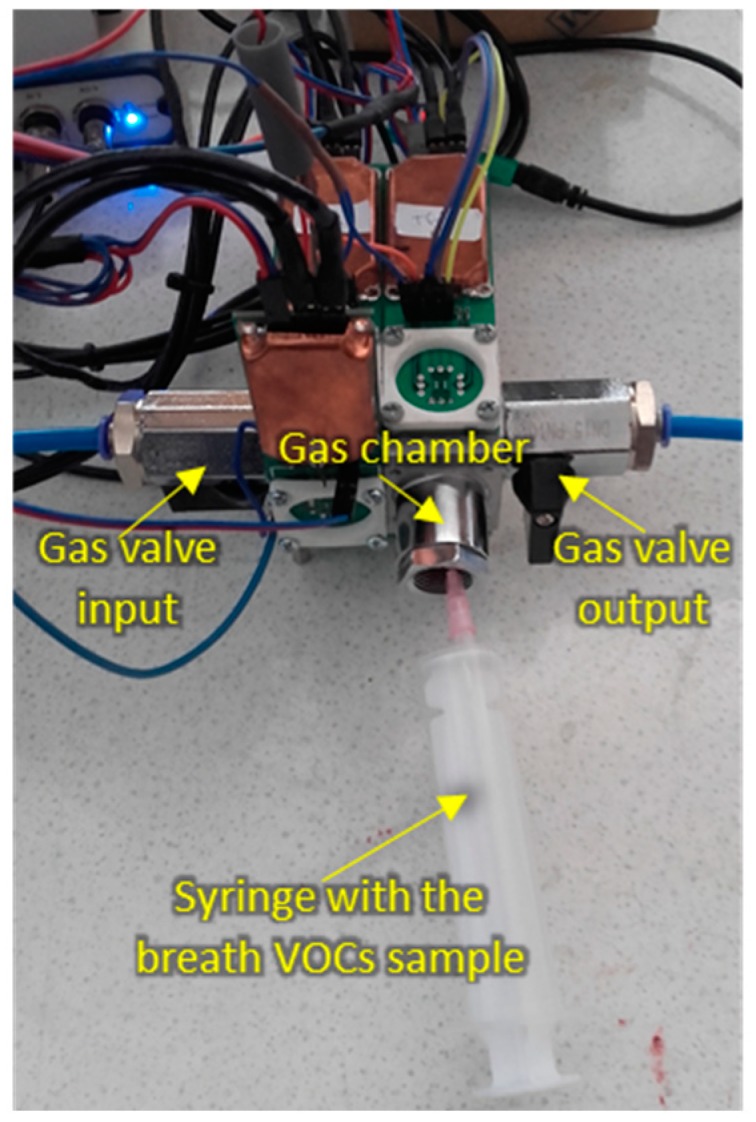
Measurement setup during breath sample injection.

The measurement setup during sample injection is shown in Figure 3.

Sensors DC resistances and AC fluctuations were acquired with separate data acquisition boards connected through USB ports to the sensors systems. DC resistances were acquired with one data acquisition board (National Instruments, USB-6216 model) at 1 Hz sampling rate, while AC fluctuations were recorded with two data acquisition boards (National Instruments, models USB-4431 for the commercial sensors and PCI-4474 for the custom WO_3_ nanowires sensor, respectively) at 20 kHz sampling frequency, each AC record consisting of 40,000 voltage samples. For estimating the power spectral densities (PSD) of the recorded AC samples, the Welch method using averages over 40 spectra was applied.

### 2.4. Data Analysis

The following features were extracted from sensors responses to each sample analyzed:

1. DC measurements:
  - **F1:** ∆R/R_0_. Relative resistance change after sample exposure. ∆R = R_min_ – R_0_ is the resistance drop after sample exposure, where R_min_ is the minimum resistance of the sensor after sample exposure and R_0_ is the sensor’s resistance in synthetic air immediately before the short vacuum step that preceded sample exposure;
  - **F2:** dR/dt. Dynamic slope of the resistance calculated at 30 s after sample injection;
  - **F3:** AUC. Area under the curve during 13 min of sample exposure

**Figure 4:**
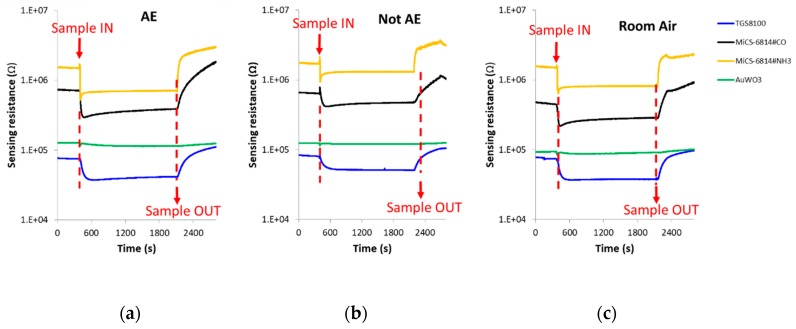

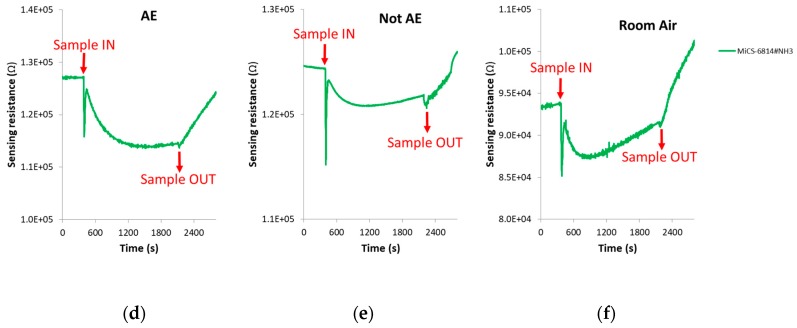
DC sensors responses in the presence of: (**a**) Breath sample of a patient with confirmed echinococcosis; (**b**) Breath sample of a control volunteer; (**c**) Room air. (**d**–**f**): Zoom of the AuWO_3_ sensor responses presented in Figure 4**a**–**c**, respectively. AE: Alveolar echinococcosis; Not AE: Control.

The spike corresponding to sample injection, which can be noted in Figure 4 where DC sensors responses is presented, was not taken into account when the DC features were calculated.

2. AC measurements:
  - **F4:** PSD value at 180 Hz, averaged over 11 points around this frequency
  - **F5:** PSD value at 400 Hz, averaged over 11 points around this frequency
  - **F6:** PSD value at 1000 Hz, averaged over 11 points around this frequency
  - **F7:** PSD value at 5000 Hz, averaged over 11 points around this frequency

These AC frequencies were selected to evenly represent the frequencies above 100 Hz, where 1/f noise is still dominant and far from any eventual drifts affecting low-frequency noise or interferences induced by power supply lines [19]. Nevertheless, no compensation of the baseline drift was realized.

In order to counteract reproducibility issues between different breath samples, for each volunteer the mean values of all features extracted from sensors responses to both breath samples provided by the same volunteer were calculated. For ensuring equal significance for all features, they were auto-scaled by subtracting the mean and dividing by standard deviation.

The Discriminant Function Analysis (DFA) algorithm was employed to build predictive models for samples classification [20]. A bootstrap validation process was performed with 10,000 repetitions and random division of the dataset in 70% of data for training and 30% of data for testing during each repetition. The classification success rate was calculated as the mean value of the percentages of correct predictions achieved during each bootstrap repetition. Sensitivity and specificity were calculated similarly to the classification success rate, as the mean value of the percentages of the sensitivity and specificity achieved during each bootstrap repetition.

## 3. Results

### 3.1. Sensors Responses

When exposed to the breath samples, the sensors responded to the overall VOCs mixture from the breath.

The change of sensors DC resistance in response to samples injection is shown in Figure 4. In this figure, the moment in which the breath sample was injected into the sensors test chamber (approximately 8 min after the beginning of the vacuum step) can be appreciated, as well as when the sample was evacuated from the test chamber (approximately 13 min after sample exposure). It can be also seen that each cross-reactive gas sensor from the array responded differently when it interacted with the breath volatiles of AE patients and controls.

**Figure 5:**
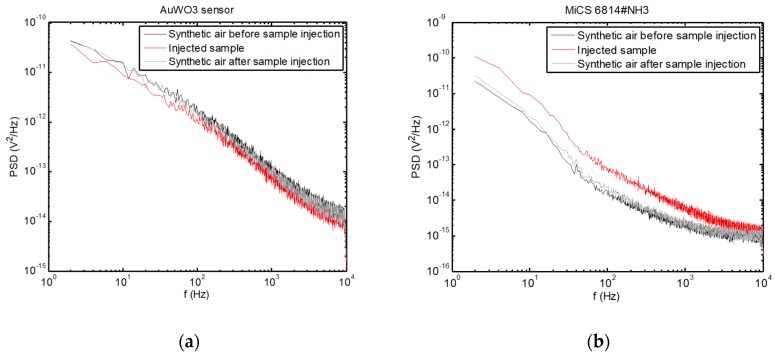
Power spectral densities of voltage fluctuations measured across the sensors exposed to a breath sample, as well as before sample measurement and after cleaning the test chamber with synthetic air. (**a**) AuWO_3_ sensor; (**b**) MiCS 6814#NH_3_ sensor.

Examples of PSD are presented in Figure 5, where the change in the PSD spectra upon injection of the breath sample can be observed, while the sensors returned to their baseline condition after cleaning the test chamber with synthetic air after sample evacuation.

**Figure 6:**
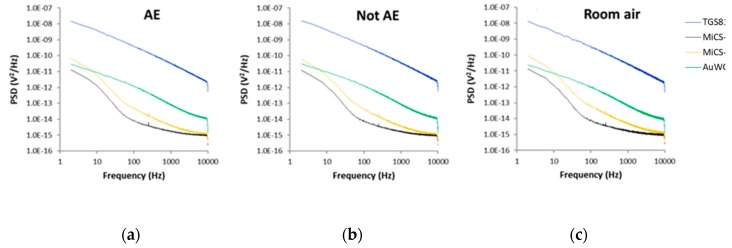
Average power spectral densities (PSD) value of sensors fluctuations recorded in the presence of: (**a**) Breath samples of all AE patients; (**b**) Breath samples of all control volunteers; (**c**) All room air samples. AE: Alveolar echinococcosis; Not AE: Control.

Differences between the PSD recorded for all sensors exposed to the breath samples of all AE patients, all controls and all room air samples can be seen in Figure 6.

In the Supplementary Materials, there are shown radar plots with unitary radius for the seven features extracted from sensors responses to each class of samples (AE, control and room air), which show the pattern differences between the various conditions.

### 3.2. Classification Results

At first, we assessed the discrimination between AE patients and controls, achieved individually by each gas sensor. The results, presented in Table 2, showed that the best discrimination was obtained by sensor S3 (63.4% classification success rate, 66.3% sensitivity and 54.6% specificity). Nevertheless, due to the high complexity of the breath composition, none of the sensors was able to achieve an acceptable discrimination when its responses were individually analyzed.

**Figure 7:**
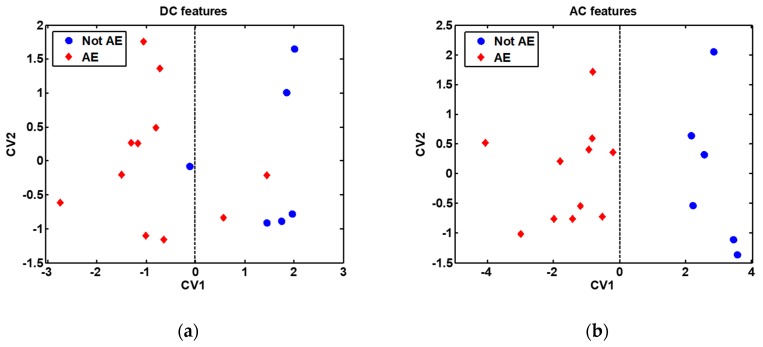
Discriminant Function Analysis (DFA) models built with all samples (without bootstrap validation), shown for visualization purpose only: (**a**) DFA model built with features extracted from the DC measurements; (**b**) DFA model built with features extracted from the AC measurements. The features used to build the DFA models are indicated in Table 2. AE: Alveolar echinococcosis; Not AE: Control. CV1: First (and most discriminative) canonical variable of the DFA model; CV2: Second canonical variable of the DFA model. The decision border is given by the vertical dashed line passing through the zero value on the CV1 axis.

In order to broaden the range of volatiles detected, the discrimination potential of the cross-reactive gas sensors array was next assessed. This analysis was performed, on the one hand, using the features extracted from the DC measurements, and on the other hand, using the features extracted from the AC measurements. The classification results obtained are presented in Table 2, while the DFA models built are presented in Figure 7. It is remarkable to note the 90.2% classification success rate, 93.6% sensitivity and 79.4% specificity obtained by the sensors array when the features extracted from the AC measurements were employed to build the discrimination model.

## 4. Discussion

### 4.1. AC versus DC Measurements

The comparison of the classification results obtained when the discrimination models were built with either the DC or AC features revealed that only the AC measurements led to a very good classification between the AE patients and controls. On the other hand, features extracted from the AC measurements were also mainly used in the case of the majority of the models that provided the best discrimination accuracies for each individual sensor in part (see Table 2).

The superior results obtained by the AC measurements can be attributed to the fact that the recorded AC fluctuations of sensors resistances are characterized by a power spectral density that is a function of frequency, and gives more information than the measurement of the DC resistance of the sensors that provides temporal information only [14]. This method was proposed more than twenty years ago and is called Fluctuation-Enhanced Sensing (FES) [19,21]. The technique utilizes low-frequency noise (flicker noise) as a source of information about the volatiles atmosphere to which the gas sensor is exposed.

Random microscopic phenomena of gas molecules adsorption–desorption generate flicker noise. Its intensity and statistical parameters (e.g., PSD) depend on these random processes. Flicker noise is susceptible to the presence of any adsorption–desorption events because fluctuation phenomena require less energy than changes of macroscopic parameters such as DC resistance. Moreover, PSD can expose the components, named Lorentzians, of the frequency’s characteristic for different gas molecules [22,23], meaning that flicker noise (its intensity and shape of PSD) can identify different gases. This is predicted by a mathematical model of adsorption–desorption noise sources [24].

The FES method requires an advanced measurement setup. The requirements necessary to evaluate the PSD of flicker noise can be reduced to a simplified analysis as presented elsewhere [25], which further reduces the necessary computations of random data to the selected frequency bandwidths. This strategy was implemented in the present study.

### 4.2. Sensors Characteristics

When sensors responses were individually analyzed for each sensor, the best discrimination accuracy was obtained by sensor S3. Alkane derivatives, such as methane, propane and iso-butane, detected by sensor S3, are recurrent oxidation products in the process of lipid peroxidation caused by highly unstable reactive oxygen species produced by the host as a result of immune responses against echinococcus infection [15,26,27]. Ethanol (detected by sensors S3 and S1) and ammonia (detected by sensors S3 and S2) are common breath metabolites. In subjects that have not consumed alcoholic beverages, ethanol is likely to come from intestinal gut flora, whereas helminth colonization is associated with increased diversity of the gut microbiota (echinococcosis is a helminthic infection) [28]. On the other hand, increased levels of ammonia in breath were associated with liver dysfunction [29], which is the body organ where the primary tumor-like lesion produced by AE is usually developed [1]. Finally, the custom sensor based on WO_3_ nanowires doped with gold nanoparticles can further favor breath VOCs detection [16,30].

Whereas the influence of the main confounding factors (smoking habit and gender) cannot be accurately estimated because of the low number of smokers (four) and males (two) that participated in the present study, the difference in the mean age of the AE patients and controls (60.6 ± 12.1% and 40.5 ± 7.4%, respectively) could have some influence on the results obtained.

## 5. Conclusions

In the present study, we have shown that by properly selecting an array of chemical gas sensors suitable for breath VOCs detection and the sensing technique employed, it is possible to discriminate with high accuracy the patients with AE from the controls group. The proposed approach provides a noninvasive, easy to use, fast and inexpensive mean for the effective diagnosis of AE at an early stage, when curative treatment can be applied.

On the other hand, the concomitant measurement of two sensors characteristics, namely DC resistance and AC resistance fluctuations, during sensors exposure to the same breath sample, demonstrated the superior performance of the AC measurement approach over the DC measurement approach.

Although performed on a small study group because of the limitations in recruiting a higher number of patients for this study due to the fact that AE is a neglected disease, it is important to note that the use of a small number of features to develop the classification models and the employment of the bootstrap validation technique for training and testing the models confers sufficient strength to the results obtained. However, the influence of possible confounding factors (smoking habit, gender and age) needs to be further assessed in a more extended study with higher homogeneity between the study groups.

## Figures and Tables

**Table 1:** Information about the volunteers included in the study.

Patient no.	Disease ^1^	Age	Gender ^2^	Smoking Habit	Medication
1 ^5^	AE	69	F	No	Sulfasalazine, Acidum, Folicum
2	Not AE	36	M	No	-
3 ^5^	AE	58	M	No	Ramipril, Nebivolol, Propafenone
4 ^4^	AE	49	M	Yes	Albendazole, Amlodipine, Bisoprolol
5 ^4^	AE	74	F	No	Enalapril, Amlodipine
6 ^5^	AE	73	F	No	Albendazole, Indapamide, Ramipril, Bisoprolol
7	Not AE	30	F	No	-
8 ^4^	AE	61	F	Yes	Albendazole, Furosemide, Spironolactone, Propranolol
9	Not AE	50	F	No	-
10 ^5^	AE	54	F	No	Albendazole, Bisoprolol, Perindopril
11 ^4^	AE	60	F	No	Albendazole, Furosemide, Spironolactone
12 ^5^	AE	75	F	No	Albendazole, Valsartan, Metformin
13	Not AE	46	F	No	Ramipril
14	Not AE	44	F	No	-
15 ^5^	AE	35	F	No	Albendazole
16 ^3,5^	AE after liver transplantation	59	F	No	Albendazole, Tacrolimus, Calcium
17	None	37	M	No	-

^1^ AE = alveolar echinococcosis. ^2^ M = male; F= female. ^3^ This patient had recurrence of AE in transplant liver. She had specific changes in CT and still positive serological tests. ^4^ Confirmed case of AE according to Brunetti’s criteria [17]. ^5^ Probable case of AE according to Brunetti’s criteria [17].

**Table 2:** Discrimination between the AE patients and controls (mean values (%) ± standard deviation)

Sensors		Features	Classification Success Rate	Sensitivity	Specificity
Individual sensors	S1	F1,F5,F6	49.1 ± 20.2%	58.7 ± 29.5%	31.2 ± 36.8%
	S2	F4,F6	49.7 ± 20.3%	57.8 ± 30.7%	34.5 ± 37.3%
	S3	F5,F6	63.4 ± 20.0%	66.3 ± 27.6%	54.6 ± 41.9%
	S4	F3,F6	45.6 ± 21.8%	49.2 ± 30.9%	37.4 ± 37.4%
Sensors array	DC measure-ments	S1: F1,F3 S2: F1,F3 S4: F2	73.4 ± 19.3%	75.9 ± 24.5%	
					64.2 ± 39.6%

	AC measure-ments	S1: F1,F4 S2: F1 S3: F1 S4: F1,F2	90.2 ± 16.4%	93.6 ± 16.8%	79.4 ± 35.8%

## Supplementary Materials

The following are available online at https://www.mdpi.com/1424-8220/20/9/2666/s1, Section S1: Features patterns.
