# Supplementary material for: Assessment of Electronic Sensing Techniques for the Rapid Identification of Alveolar Echinococcosis through Exhaled Breath Analysis
Source: Sensors (Basel). 2020 May 7;20(9):2666. doi: 10.3390/s20092666 (PMC7249121; doi:10.3390/s20092666)
Supplement: Supplementary file 1 [file sensors-20-02666-s001.pdf]

Supplementary Material

Assessment of Electronic Sensing Techniques for the Rapid Identification of Alveolar Echinococcosis through Exhaled Breath Analysis

Andrzej Kwiatkowski [1], Tomasz Chludziński [1], Tarik Saidi [2,3], Tesfalem Geremariam Welearegay [4,5], Aylen Lisset Jaimes-Mogollón [6,7], Nezha El Bari [3], Sebastian Borys [8], Benachir Bouchikhi [2], Janusz Smulko [1] and Radu Ionescu [5,*]

1 Department of Metrology and Optoelectronics, Faculty of Electronics, Telecommunications and Informatics, Gdańsk University of Technology, Gdańsk 80233, Poland; Andrzej.Kwiatkowski@pg.gda.pl (A.K.); tomekchlud@gmail.com (T.C.); janusz.smulko@pg.edu.pl (J.S.)
2 Sensor Electronic & Instrumentation Group, Faculty of Sciences, Department of Physics, Moulay Ismaïl University of Meknes, B.P. 11201, Zitoune, 50050 Meknes, Morocco; saidi.tareq@gmail.com (T.S.); benachir.bouchikhi@gmail.com (B.B.)
3 Biotechnology Agroalimentary and Biomedical Analysis Group, Faculty of Sciences, Department of Biology, Moulay Ismaïl University of Meknes, B.P. 11201, Zitoune, 50050 Meknes, Morocco; n.elbari@umi.ac.ma
4 Department of Electronics, Electrical and Automatic Engineering, Rovira i Virgili University, Tarragona 43007, Spain; gem.tesfa@gmail.com
5 The Ångström Laboratory, Division of Solid State Physics, Department of Materials Science and Engineering, Uppsala University, Uppsala 75121, Sweden
6 GISM Group, Faculty of Engineering and Architecture, University of Pamplona, 543050 Pamplona, Colombia; lissetjaimes@gmail.com
7 Department of Chemical Engineering, Complutense University of Madrid, 28040 Madrid, Spain
8 University Centre of Maritime and Tropical Medicine, Gdynia-Redlowo 81519, Poland; sebek07@op.pl
* Correspondence: radu.ionescu@angstrom.uu.se; radu.ionescu@urv.cat

S1. Features patterns

The figures below present the radar plots with unitary radius for the seven features extracted from sensors responses to each class of samples (AE, control and room air). The projected samples were randomly selected.

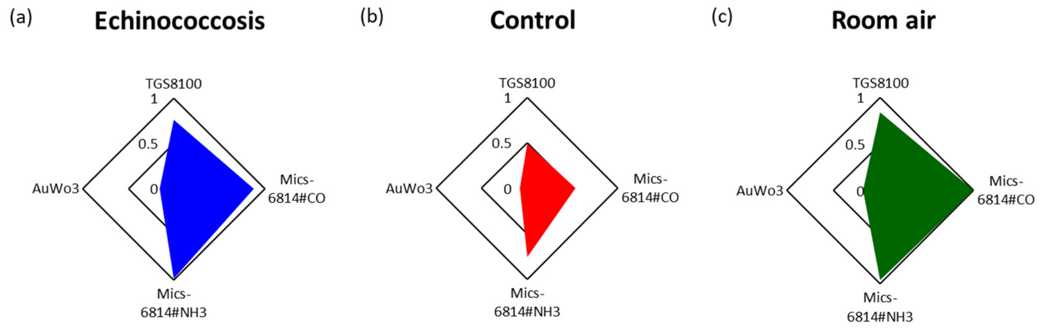

Figure S1. Radar plots expressed by F1 feature

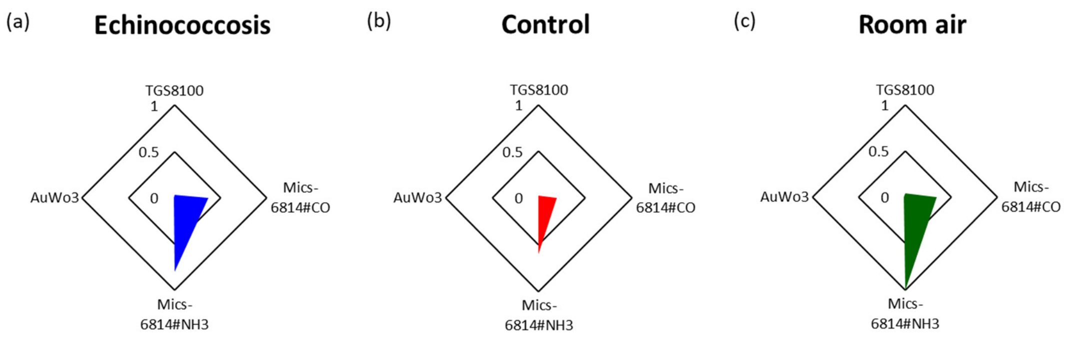

Figure 2. Radar plots expressed by F2 feature

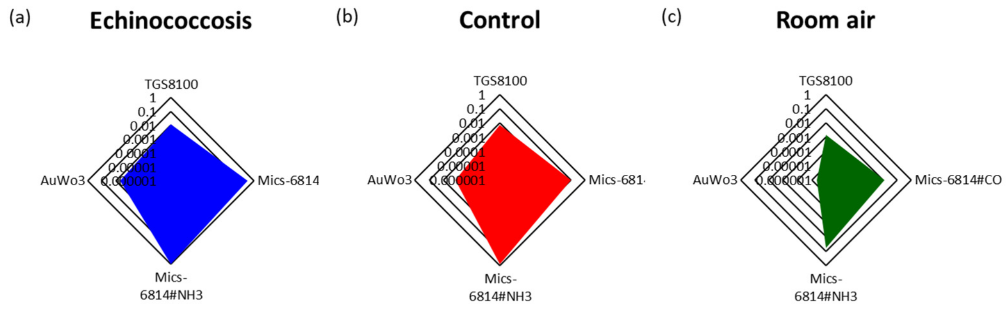

Figure S3. Radar plots expressed by F3 feature

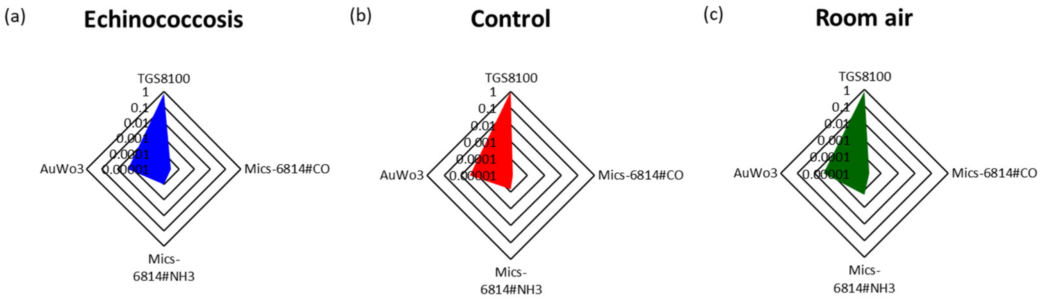

Figure S4. Radar plots expressed by F4 feature

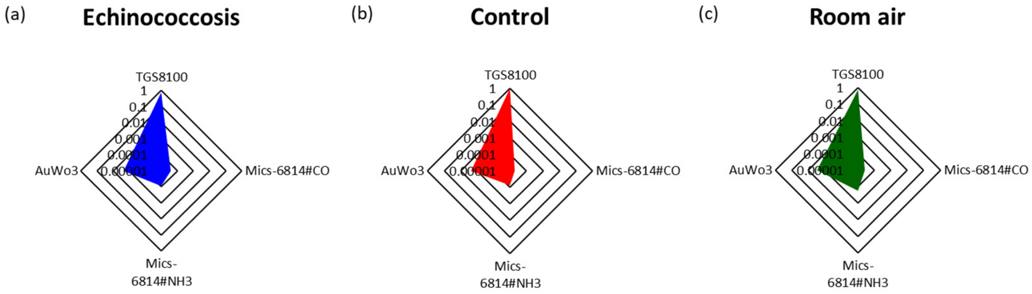

Figure S5. Radar plots expressed by F5 feature

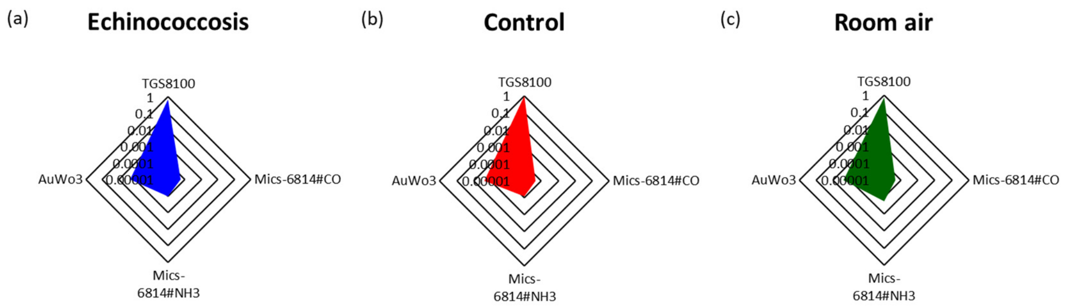

Figure S6. Radar plots expressed by F6 feature

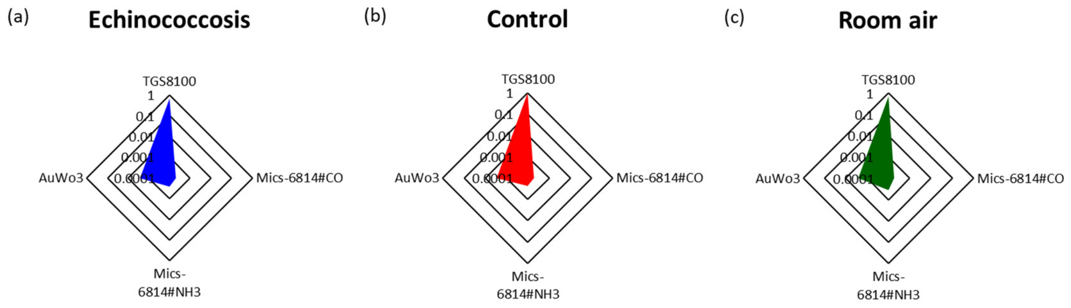

Figure S7. Radar plots expressed by F7 feature
